# Relationship Health and Intimate Partner Violence in Integrated Primary Care: Individual Characteristics and Preferences for Relationship Support across Risk Levels

**DOI:** 10.3390/ijerph192113984

**Published:** 2022-10-27

**Authors:** Dev Crasta, Cory A. Crane, Nicole Trabold, Robyn L. Shepardson, Kyle Possemato, Jennifer S. Funderburk

**Affiliations:** 1 Center of Excellence for Suicide Prevention, Department of Veterans Affairs, Canandaigua, NY 14424, USA; 2 Department of Psychiatry, University of Rochester Medical Center, Rochester, NY 14642, USA; 3 College of Health Science and Technology, Rochester Institute of Technology, Rochester, NY 14623, USA; 4 Center for Integrated Healthcare, Syracuse VA Medical Center, Syracuse, NY 13210, USA; 5 Department of Psychology, Syracuse University, Syracuse, NY 13244, USA

**Keywords:** primary care, integrated primary care, intimate partner violence (IPV), marital conflict, couple communication

## Abstract

This study explores differences in characteristics and relationship treatment preferences across different levels of intimate partner violence (IPV) among Veterans Affairs (VA) primary care patients. In Fall 2019, we sent a mail-in survey assessing relationship healthcare needs to N = 299 Veterans randomly sampled from 20 northeastern VA primary care clinics (oversampling female and younger Veterans). We compared those reporting past year use or experience of physical/sexual aggression, threats/coercion, or injury (Severe IPV; 21%), to those only reporting yelling and screaming (Verbal Conflict; 51%), and denying any IPV (No IPV; 28%). Participants across groups desired 2–6 sessions of face-to-face support for couples’ health and communication. No IPV participants were older and had preferred treatment in primary care. The Verbal Conflict and Severe IPV groups were both flagged by IPV screens and had similar interest in couple treatment and relationship evaluation. The Severe IPV group had higher rates of harms (e.g., depression, alcohol use disorder, relationship dissatisfaction, fear of partner) and higher interest in addressing safety outside of VA. Exploratory analyses suggested differences based on use vs. experience of Severe IPV. Findings highlight ways integrated primary care teams can differentiate services to address dissatisfaction and conflict while facilitating referrals for Severe IPV.

## 1. Introduction

Romantic relationship health–defined as strong connections between partners as a couple (e.g., satisfaction, honesty, open communication) and mutual support of each partner as an individual (e.g., emotional support around stressors, equal and respectful approach to conflict management) [1,2,3]—can be an asset to treatment adherence, physical health, and quality of life [1,2]. However, relationship conflict and intimate partner violence (IPV)–defined as physical violence, sexual violence, stalking, and psychological aggression between romantic partners [4]—can have significant negative impacts on physical and mental health [5,6]. This would suggest supporting relationship health can be a valuable component in comprehensive healthcare, a position affirmed by recommendations to screen for IPV and refer to support services made by both the United States Preventive Services Task Force (USPTF [7]) and Centers for Disease Control (CDC [8]). The Department of Veterans Affairs (VA), the largest integrated healthcare system in the United States, has demonstrated that such screening efforts are feasible and can be scaled up at hospital-wide or national levels [9]. However, screening is only one aspect of preventive healthcare, and commentators have highlighted the potential for primary care to play an active role in preventing IPV at a population level remains largely untapped [10].

Additional opportunities to expand IPV prevention beyond screening are supported by the rapid growth of integrated primary care settings over the last decade across federal hospital systems (e.g., VA, Department of Defense) and large community clinics [11]. Integrated primary care describes a range of models that have a primary care team collaborate with trained behavioral health providers. These embedded behavioral health providers assist the primary care teams with further assessment, the provision of brief interventions when appropriate, and can act as a bridge to specialty mental health services if needed [11]. Guided by the increasing number of integrated primary care settings and the prevention health framework, the current study explores the unique characteristics of primary care patients and preferences for IPV prevention efforts for these patients at different levels of risk.

### 1.1. Applying a Levels of Prevention Framework to IPV in Primary Care

In this study, we apply to IPV a levels of prevention framework [12], which focuses on differentiating approaches across a range of risk from Primary Prevention (health promotion to prevent the emergence of a problem in individuals who are not currently experiencing it), to Secondary Prevention (screen individuals who experience problem at an early stage to address/prevent harm), to Tertiary Prevention (treat individuals already impacted by a problem to stop further harm). This framework can guide intervention development as patients typically have differing needs at each level. Organizing interventions by these levels facilitates efficient stepped care whereby patients can be served according to their needs to conserve healthcare resources.

Complete absence of IPV behaviors on a primary care screen immediately identifies patients at low risk (i.e., no IPV reported) and appropriate for Primary Prevention approaches. The CDC offers a toolkit of IPV Primary Prevention strategies for communities. Although some focus on institutional resources that do not apply to medical settings (e.g., financial and legal support), two that may be appropriate for integrated primary care are “Teach Safe and Healthy Relationship Skills” and “Disrupt the Developmental Pathways towards Partner Violence” [8]. As both approaches focus on psychoeducation, they can feasibly be integrated into existing patient education by existing primary care providers or offered by embedded behavioral health providers in those teams.

Secondary Prevention is defined by efforts delivered following a positive IPV screen but prior to harm. Unfortunately, the CDC toolkit’s single strategy addressing screening, “Support Survivors to Increase Safety and Lessen Harm” assumes referral to intensive services is the only option for a positive screen (blending Secondary and Tertiary Prevention). CDC has described efforts to differentiate psychological aggression between common expressions of anger or verbal outbursts and “psychological abuse” patterns that cross a threshold of harm [4]. Although CDC efforts are ongoing, one practical way to create a Secondary Prevention category is by using the items in many IPV screens that distinguish between “verbal conflict” behaviors such as screaming and insults from other more severe forms of IPV [7]. This distinction would align with the epidemiological IPV literature, where yelling and insults are classified as “minor psychological aggression” that are noted to occur in >60% of individuals in population samples while all other types of IPV–including coercive and controlling behaviors identified as “severe psychological aggression”—are reported by <20% of individuals [13]. Verbal conflict is an appropriate category for differentiating treatment as it can be addressed by a wider range of services. Under the name “conflict management” or “communication skills training,” verbal conflict behaviors are frequently addressed by relationship skills education programs [14,15] and many behavioral couple therapies [16]. The skills are often concrete cognitive behavioral strategies that can easily be incorporated into a behavioral health provider’s skillset.

All other “severe IPV” behaviors, such as psychological control/coercion, stalking, physical violence, and sexual violence, can be classified as requiring Tertiary Prevention as they are less common and have greater potential for physical and psychological harm Treating severe IPV will likely require referral to specialty care as IPV can have different patterns at a couple level, each requiring separate specialized skillsets. One way to distinguish patterns is by “directionality,” as individuals might solely use IPV on their partner, solely experience IPV from their partner, or may have complex “bidirectional IPV” relationships where both partners use IPV. Another way to synthesize this information when making referrals is by attending to power and control that relationship [17]. In patterns where one partner uses IPV as part of a pattern of control, guidelines suggest partners should be treated separately. Many community agencies offer efficacious interventions both for those who experience IPV [18] and use IPV [19]. A larger portion of couples are in “situational couple violence” relationships, primarily bidirectional patterns where violence reflects a lack of control of emotions in both partners. In these cases, couples can be treated together, but further evaluations for severity may be needed to differentiate between traditional couple therapy for those with low severity IPV [20] and specialized conjoint IPV treatments for couples with higher severity situational IPV [21,22]. Couple therapy clinics, court-mandated programs, and shelters routinely assess for directionality, control patterns, and injury potential at intake, but by considering these elements at the moment of identification, primary care can facilitate more accurate referrals.

### 1.2. Optimizing Prevention Efforts in Integrated Primary Care Settings

Although a handful of secondary prevention IPV programs have been developed for primary care [23], the above research highlights that integrated primary care settings are ideally suited to provide a larger continuum of preventative approaches to IPV. Potential roles range from psychoeducation and discussion of safety by all providers, simple skills training by an embedded behavioral health provider, to differentiating referrals for high-risk clients. To assist in optimizing patient engagement and satisfaction in these variety of intervention options, it is important to consider patient preferences [24,25] for attributes (e.g., number of appointments) and foci (i.e., relationship concerns addressed). Furthermore, attending to preferences increases initial utilization of a service [26] and lowers dropout rates after engagement [27,28]. Past work has identified relationship concerns in low-income families [29] and patients seen in intensive couple therapy clinics [30], there has been no prior work on preferred relationship concerns to address in primary care and no prior work on preferred attributes for relationship treatments in any setting.

### 1.3. The Current Study

Due to the lack of existing research, the present study aims to guide further development of IPV interventions suitable for the all patients along the risk continuum served in healthcare systems by (1) characterizing groups across the IPV risk continuum with re-spect to demographics, psychological disorders, and relationship health and (2) examin-ing preferences for relationship support across the IPV risk continuum. The study focuses on sample of men and women receiving primary care services in VA, a system that al-ready embraces an integrated primary care setting and excels in the screening and referral model of IPV treatment but may benefit from expanding the role of primary care.

## 2. Materials and Methods

Study measures were included in a larger cross-sectional mail survey assessing relationship functioning and IPV in VA primary care. All study procedures were approved by the Syracuse VA Institutional Review Board (IRB#1420784).

### 2.1. Study Design and Recruitment

Veterans were recruited from three Veterans Affairs Medical Centers and their associated community-based outpatient clinics in Central and Western New York in August 2019 (20 clinics total). We used the electronic medical record (EMR) to identify Veterans meeting the following inclusion criteria (1) age 18–85; (2) utilized primary care services in calendar year 2018 and (3) demographic information suggested being in a relationship. We excluded Veterans who (1) did not have complete mailing address in the EMR or (2) had a diagnosis of major neurocognitive disorder, delusional disorder, or severe/profound intellectual disability, to improve the likelihood of accurate survey completion. We then randomly sampled a group of 1,500 Veterans with a goal of achieving a target sample of 300 respondents to be sufficiently powered for latent variable models of IPV typologies in the larger study. In order to capture a diversity of IPV behaviors in a small sample, we oversampled Veterans below the age of 55 (4:1, or 1200 Veterans below 55) as IPV prevalence declines after age 55 [31]. Similarly, we oversampled female Veterans (1:1 ratio; or 750 female Veterans) as IPV typologies often differ by gender [17].

We mailed each veteran a recruitment letter, information sheet explaining the study, and survey measures in August 2019. Interested participants could mail the completed survey back using pre-stamped, self-addressed envelopes in return for a $20 incentive. Of 317 participants who returned surveys (21% response rate), three did not provide sufficient demographic data and 15 were not in a current romantic relationship, leaving a final sample of 299 participants.

### 2.2. Measures

#### 2.2.1. Intimate Partner Violence

The Conflict Tactics Scale Short Form (CTS2S [13]) is a brief version of the Revised Conflict Tactics Scale (CTS-2 [32]), often regarded as the gold standard of IPV assessment. The CTS2S assesses mild and severe behaviors for each of the five dimensions of the CTS-2 including Negotiation, Psychological Aggression, Physical Aggression, Sexual Aggression, and Injury. For each item, participants report both whether they have used that behavior on their partner or experienced that behavior over the previous year. Individuals are classified as experiencing or using severe IPV if they reported any past year Physical Aggression (i.e., hitting or attacking), Sexual Aggression (i.e., sexual coercion or rape), Injury (i.e., physical harm as the result of a conflict), or Severe Psychological Aggression (i.e., threats or property destruction). This reflects the CTS2 cutoffs used to standardize IPV screening measures in VA [33,34]. Following the recommendations of the scale authors, frequencies from the mild Psychological Aggression item was aggregated to their midpoint to obtain an approximate count of verbally hostile behavior (i.e., screaming at or insulting a partner) over the previous year [13].

#### 2.2.2. Mental Disorders

Participants also completed a range of well-validated measures to assess clinical conditions known to be associated with IPV. The Patient Health Questionnaire-9 (PHQ-9 [35]) demonstrated high internal consistency in the sample (α = 0.90) and was used to assess depression (cutoff of ≥10) and recent thoughts of suicide or self-harm (using any positive scores on the ninth item [36]). Probable posttraumatic stress disorder (PTSD) was assessed using the PTSD Checklist (PCL5 [37]; α = 0.97) using a cutoff suggested for general health settings (scores ≥ 31). Potential alcohol misuse was identified using scores ≥ 8 on the Alcohol Use Disorder Identification Test (AUDIT [38]; α = 0.85).

#### 2.2.3. Relationship Health Screens

Relationship functioning was assessed using the four-item screening version of the Couples Satisfaction Index (CSI-4 [39]). The scale demonstrated excellent internal consistency in the sample (α = 0.96) and we used the optimal cut-off score of <13.5 to identify distressed participants. The Extended Hurt-Insult-Threaten-Scream (E-HITS [33]) scale is a five-item measure of IPV experience that was developed and validated for screening in primary care settings. We used the suggested cutoff score of ≥7 to identify Veterans who would be classified as experiencing a level of IPV requiring further care in a primary care setting. The 5-item Danger Assessment (DA-5 [40]) captures the larger context for IPV (e.g., escalating violence; partner owns a weapon) to predict likelihood of future injuring or lethal assaults. The Women’s Experience of Battering Scale (WEB [41]) explores the psychological experience of power and fear.

#### 2.2.4. Preferences for Treatment Attributes and Foci

Participants’ preferences were assessed through a series of forced-choice items asking participants, “If you had to pick only one [Type/Format/Location/Length] for help with relationship concerns, which would you prefer?” Following guidance from the preference literature [24], we also provided non-technical descriptions of Type and Format terms (see Table 1). We allowed participants to indicate “No Preference” to reduce the likelihood of random responding in the absence of a strong opinion. Preference for treatment focus was assessed through a series of 14 items asking participants “Rate how likely you would be to attend help offered within the VA for each Relationship Concern listed below” Each concern was rated on a 1 (*Very Unlikely*) to 5 (*Very Likely*) Likert scale. Responses were dichotomized so that scores of 4 (*Likely*) and 5 (*Very Likely*) were coded as “Likely to attend.” Each concern area included a non-technical description of how treatment would address that concern (see Table 1).

### 2.3. Analytic Strategy

#### 2.3.1. Defining Groups

Data were analyzed using SPSS 22. To represent a continuum of risk for different levels of prevention, we stratified our sample into three groups. The “Severe IPV” group (i.e., those targeted for Tertiary Prevention) included 63 respondents (21% of the sample) who reported using or experiencing physical IPV, sexual IPV, severe psychological IPV, or injury in the previous year on the CTS2S. A second “Verbal Only” group (i.e., those appropriate for Secondary Prevention) included the 152 respondents (51% of full sample) who reported using or experiencing mild psychological aggression (e.g., yelling or screaming) but denied all other IPV behaviors. The remaining 84 respondents (28% of full sample) denied using or experiencing any form of IPV over the previous year were classified as “No IPV” (i.e., appropriate for Primary Prevention activities).

#### 2.3.2. Comparisons between Groups

Although group membership could be conceptualized as part of an ordinal sequence, ordinal tests yielded too many significant results that were not clinically meaningful (i.e., small monotonic trends reflecting higher endorsements of all items among high-risk groups). Therefore, we used a more conservative approach and evaluated overall group differences using initial χ^2^ tests for independence (for categorical outcomes) or analysis of variance (for continuous outcomes). Significant results were followed by post hoc tests for each pairing of groups (χ^2^ for categorical outcomes; Tukey’s Honest Significant Difference for continuous outcomes) to identify homogenous subsets. Significance was set at *p* < 0.05 for all tests. For characteristics that were significantly higher in the IPV group than the other two groups, we then conducted sub-analyses comparing individuals that reported solely using IPV, solely experiencing IPV, and both using/experiencing IPV. Although these analyses are underpowered for significance testing, we report differences when the highest subgroup has >20% endorsement than the lowest subgroup.

## 3. Results

### 3.1. Sample Characteristics

See the first column of Table 2 for characteristics of the sample. Reflecting our sampling strategy, our respondents were younger and had a larger percentage of women than typical veteran samples but were otherwise representative of Veteran demographics in the recruitment region with respect to race, ethnicity, and marital status. Among the 63 participants reporting past-year severe IPV behaviors in their relationship (i.e., coercive psychological IPV, physical or sexual violence, and/or injury), 35% exclusively experienced severe IPV, 22% exclusively used severe IPV, and 43% reported bidirectional severe IPV. In contrast, a vast majority of the 152 Veterans reporting exclusively Verbal Conflict in their relationship reported that it was bidirectional (88%) with smaller numbers reported that they exclusively screamed or yelled at their partners (5%) or that their partners exclusively screamed and yelled at them (7%) over the previous year.

### 3.2. Demographic and Relationship Differences by Group

As seen in the remaining columns of Table 2, respondents denying IPV tended to be older than those reporting Verbal Conflict or Severe IPV in their relationship. Participants in Severe IPV Relationships also more frequently screened positive for Depression and alcohol misuse. Groups were otherwise similar across all demographic and clinical categories. Participants in severe IPV relationships reported a higher frequency of using verbal conflict behaviors than the Verbal only group but did not report significantly higher experience of IPV by their partners, highlighting the difficulty of defining a clear cutoff for Verbal Conflict. Since the E-HITS Experience Screen is sensitive to verbal conflict, endorsement increased across all three groups. In contrast, the WEB measure of control and the DA-5 measure of injury risk factor more clearly identified the highest risk group.

Given the heterogeneity between different dyadic patterns in the Severe IPV group, we conducted follow-up descriptive analyses of the relationship health screens in those groups. Although underpowered to detect significant differences, this provided some insights into group divisions. Specifically, the E-HITS detected 43/49 (88%) of the individuals who experience severe IPV in either one-way or bidirectional IPV relationships. However, the E-HITS also flagged 10/14 (71%) individuals who only used severe IPV behaviors, suggesting this screen’s attention to insulting/screaming might also detect prominent verbal conflict behaviors that their partners used out of fear. In contrast, individuals who solely used severe IPV were rarely flagged by the WEB measure of Fear (2/14 (14%)) and did not report any risk factors for injury by their partner (0/14), suggesting greater specificity. Depression was largely similar across IPV subgroups. However, a full 43% of those who solely used IPV met AUDIT thresholds for alcohol use disorder while only 22% of those reporting bidirectional IPV and 9% of those experiencing IPV crossed alcohol threshold lines.

### 3.3. Treatment Preferences

Attribute preferences across the sample and by subgroup can be found in Table 3. Notably, all three groups were largely similar in preferring face-to-face treatment in a couples format ranging from 2–6 visits in length. However, the preference for couples treatment was much stronger in the two IPV groups than the other No IPV Group. Furthermore, significant differences emerged in preferred setting, with participants in the Severe IPV group demonstrating a stronger preference for care Outside VA than individuals in the No IPV group while the other groups preferred to stay within VA primary care. Sub-analyses within the severe IPV group suggested that the preference for non-VA care was strongest among those solely experiencing IPV (50%) while it was weaker among those who solely use IPV (36%) or who are in bidirectional IPV relationships (22%).

Likelihood of attending treatment for different concerns can be found in Table 4. Treatment addressing physical health (current and future), broad themes of relationship improvement, and communication had high rates of endorsement across the sample and were similar across groups. The specific topic of “Relationship Evaluation” had a higher rate of endorsement among individuals reporting Severe IPV in their relationship than No IPV, with individuals in Verbal Conflict only relationships had interest somewhere in the middle. Topics that separated the Severe IPV group from the Verbal Conflict only group included “Improving the Home for my Children,” “Improving Safety in the Relationship,” and “Is it time to end?” To understand this effect further, we calculated descriptive statistics for these three topics and found only one (7%) of the 14 participants who solely used IPV had an interest in “Improving Safety in the Relationship,” whereas this focus was more attractive to in individuals in bidirectional IPV relationships (37%) and or those who solely experienced IPV (32%). Interest in “Improving the Home for my Children” and “Is it time to end” were more similar across these groups (rates of participants likely to attend within 15% of one another across all three groups).

## 4. Discussion

Understanding the complexity of IPV presentations and patient preferences can help to enhance primary care’s contributions to IPV prevention at a population level. The present study offers considerations for healthcare systems differentiating treatment across multiple levels of risk. First, in taking the novel approach of sorting respondents into a continuum, the study provides novel insights into differences between groups at each stage. Furthermore, the present study is the first to examine preferences for relationship support in primary care across an IPV risk continuum. The results can guide integrated primary care teams in selecting between existing services to respond to patient preferences and can inform intervention development to improve prevention services across the risk continuum.

### 4.1. Patient Characteristics and Supporting Relationships at a Population Level

The largest portion of our primary care Veteran sample reported they experienced Verbal Conflict behaviors in a bidirectional pattern without more severe IPV behaviors. This group was similar to the higher risk group in its young age and desire for relationship services but did not differ from the No IPV group with respect to mental health concerns (i.e., depression, PTSD, alcohol use disorders) or relationship harms (i.e., dissatisfaction and fear of partner). This is consistent with the high prevalence of Mild Psychological Aggression in community studies [13] and supports addressing verbal conflict behaviors as a “Secondary Prevention” category of IPV (i.e., disorder present, but prior to harms). Furthermore, approximately 1/3rd of individuals who denied all forms of IPV in their relationship still reported relationship dissatisfaction, fear of their partner, or risk factors for escalation to injury, suggesting that relationship problems are still experienced in the “low-risk” group and highlight the potential importance of Primary Prevention. These findings are consistent with previous studies of VA primary care that suggest a majority of partnered Veterans report some form of troubled relationship [42].

One considerable challenge highlighted by our findings is the difficulty of classifying risk by focusing on the experience of IPV alone, as is recommended by both the USPSTF guidelines (i.e., screen women of childbearing age) and CDC guidelines (i.e., identify and “Support Survivors”). Consistent with its Secondary Prevention function, the E-HITS experience screen detects Verbal Conflict and more severe IPV behaviors but did not sufficiently distinguish between groups and even detected those who were the sole partners using severe IPV. At the same time, extensive measures such as the full CTS2 (78 items) or even the briefer CTS2S (20 items) are infeasible for routine use in primary care. The simplest step that can be taken is modifying reporting to distinguish between individuals with positive screens based on screaming/insults alone vs. all other IPV behaviors–as is currently done in the scoring system for the Screener for Clinically Significant IPV [43]. Alternatively, our data suggests measures focused on the power/control and injury risk also increase specificity to differentiate those who experience Severe IPV from Verbal Conflict Only. This supports the potential value of multi-stage screening process as is currently used in VA [9], as “second stage screenings” will not burden patients unnecessarily but will both differentiate between groups and provide immediate guidance on whether referral to conjoint treatments is contraindicated. The most resource intensive option would be to routinely screening for IPV use to detect participants who exclusively use IPV and to distinguish between one-way patterns. While routinely administering a second screen will increase time for both patients and providers, the last decade has seen a growing number of brief IPV use screens that may meet the needs of clinical settings [44].

### 4.2. Treatment Preferences and Directions for Intervention Development

Individuals who denied IPV showed a distinct preference for treatment in primary care, suggesting it may be an ideal site for Primary Prevention activities. Furthermore, respondents across the continuum expressed an interest in face-to-face, 2–6 session treatments addressing physical health in the relationship, highlighting that a large proportion of patients would be willing to utilize relationship supports addressing these themes. Regarding potential Primary Prevention interventions, reviews highlight a wide range of couple-based programs that have been developed to manage chronic health conditions (e.g., dementia, heart disease; [45]) and promote health behavior changes (e.g., diet, exercise [46]). By teaching partners to collaborate around health problems, these programs tend to show comparable reductions to disability as individual education along with secondary mental health and relationship benefits (e.g., reducing depression or relationship distress). Although they would be consistent with an integrated behavioral health provider role, many of these programs are designed to be delivered by a range of professionals in integrated healthcare teams (e.g., dieticians, health behavior coaches), allowing relationship support to be integrated into routine healthcare activities without burdening any particular provider.

Individuals who solely report Verbal Conflict have the widest range of available interventions, including skills education [14] and a handful of interventions developed for primary care [23]. The present results offer some guidance for selecting between these options as these individuals share the strong preference for conjoint treatments and relationship evaluation like higher risk groups while reporting a comparable preference to receive services in primary care as lower risk groups. One program that balances these considerations is the Relationship Checkup [47], a 2–3 session couple-based assessment-feedback intervention that helps couples evaluate their relationship strengths and concerns and identify concrete steps to work on their challenges (including further referral, if needed). Although originally developed for trained couple therapists, the program has been recently abbreviated to a 30-min session version designed to be delivered by co-located behavioral health providers in primary care clinics [48] and a “before baby” checkup for obstetrics clinics [49]. Notably, efficacy data suggests an “annual checkup” model of periodic two 2-session Checkups can lead to comparable improvements as more intensive programs [50].

At the Tertiary Prevention level, participants implicitly recognize the need for specialty treatment, expressing increased preference for care in behavioral health or non-VA clinics. At the same time, this group reported stronger preferences for conjoint treatments. Therefore, it will be important to evaluate fear/control and injury potential at the point of referral (e.g., using a two-stage strategy discussed above) to divert high risk couples to individual services while allowing lower-risk couples to benefit from appropriate conjoint IPV services [20,21,22]. Given the elevated association of alcohol dependence and severe IPV use, it may be important to reassess drinking and direct patients to “integrated IPV programs” that show efficacy at reducing both IPV use and substance misuse [51]. While diversion to these services increase safety, no single safety concern was desired by a majority of individuals in this high-risk group. One approach consistent with an integrated behavioral health skillset is using motivational interviewing to address ambivalence (e.g., disappointment at ineligibility for conjoint treatment; disinterest in addressing alcohol) and to link the options discussed to the strongest motivation for that individual (e.g., linking integrated treatment to health benefits that are important to a patient). A review of motivational interviewing for IPV suggest that only 1–2 sessions are sufficient to increase referral engagement and reduce subsequent dropout [52].

### 4.3. Limitations

Conclusions drawn from these findings are constrained by the following limitations. Foremost, even though our purposive sampling approach created a sample that demographically is demographically closer to the general population, our sample is still exclusively composed of Veterans and future replication in civilian samples may be needed to generalize to non-VA primary care settings. Similarly, although our mail-in survey had comparable response rates to other mailed surveys of Veterans [53], it is possible that response biases reflect participants that are more invested in relationship support than the general population. While this may lend credence to findings about undesirable options (e.g., it is quite likely that the general population would be even less likely to attend 13+ session treatments focused on legal risk than the current sample), it might overestimate actual utilization rates of desirable options. A third limitation is that our analyses did not address other potential variables that may influence treatment preferences. Prior work suggests gender impacts the preferred treatment focus [30], but it is also possible differences between risk groups (e.g., age, depression, PTSD, low satisfaction) contributed to the varied treatment preferences observed in the current sample. Studies exploring these demographic factors as predictors of treatment preferences in their own right may lead to further tailoring of relationship resources by population (e.g., resources specific to Women’s Health Clinics). A fourth limitation is that the survey did not assess psychopharmacological medication use, which prior research suggests is higher among those who have experienced IPV [54]. Future studies may be able to clarify the role of psychopharmacological interventions in addressing the sequelae of IPV by examining medication use as a covariate and potential treatment option. Another limitation is that data collection occurred prior to the 2019 Coronavirus Disease pandemic. The dramatic increase in telehealth usage during the pandemic may have increased the acceptability of phone and telehealth options since our survey. A final limitation is the CTS2S that was used to classify relationship groups. Although validated against its longer counterpart, the CTS2S has lower sensitivity than the more exhaustive CTS-2 and therefore may underestimate the presence of severe IPV in this sample [13]. Future research using the full CTS-2 may provide clearer delineation along the IPV-risk continuum.

## 5. Conclusions

The present findings highlight important avenues for integrated primary care teams to expand their population-level prevention of IPV in ways that are responsive to the diversity of patient presentations and preferences. First, the results highlight the high prevalence of relationship dissatisfaction and verbal conflict among partnered patients and the unique position of verbal conflict as a potential Secondary Prevention target. Secondly, the results highlight the limits of current screening practices to differentiate these groups and suggest possible avenues for expansion. Finally, preference data can guide the selection and development of services that are attractive to patients at different levels across the continuum, including incorporating romantic partners into health-oriented programming for all patients, having behavioral health providers offer assessment interventions for verbal-conflict couples, and offering motivational interviewing to help high-risk patients connect with services appropriate for their needs.

## Figures and Tables

**Table 1:** Brief, Non-Technical Descriptions provided to Participants.

Prompt
** Response Option—**Response Description
**If you had to pick one TYPE of help for relationship concerns, which would you prefer?**
**Couple—**Meetings with you, your partner/spouse, and a health professional. Meeting content focuses on you learning about your relationship and how to work together to improve.
**Individual—**One-on-one meetings with a health professional. Meeting content focuses on you learning about your relationship and how to improve it.
**Virtual Health Coach—**Use a virtual health coach to learn about relationships and how they might be affecting your health and what resources there might be.
* Group—Meetings with other Veterans with relationship concerns, led by a health professional. Meeting content focuses on group members sharing their personal experiences and trading tips for how to improve their relationships.
* Class—Meetings with other Veterans with relationship concerns, led by a health professional. Meeting content focuses on the group leader teaching group members about relationships and demonstrating skills to improve it. Group members do not share much about their personal experiences.
**If you had to pick one FORMAT for help with relationship concerns, which would you prefer?**
**Face-To-Face—**I meet in person (face-to-face) with a health professional.
* Telephone—I speak with a health professional on the telephone.
* Video Chat—I speak with a health professional using a secure online system
**Internet—**I complete online treatment modules/courses through a website from my own home
**Mobile App—**I use tools within a mobile app from my own home.
**Self-Help Materials—**I read paper handouts or treatment manuals on my own.
**How likely would you be to attend help focused on each concern?**
**Improving Our Relationship—**Making a relationship better and/or stronger
**Anger Management—**Learning how to better control anger when we argue
**Learning How to Be Calm—**Learning skills to stay calm when talking about difficult issues
**Improving Couple Communication—**Reviewing strategies to improve communication
**Improving Safety in a Relationship—**Identifying ways to reduce any potential harm that may happen
**Improving the Home For My Children—**Learning to provide better support or model good behaviors
**Reducing Legal Risk—**Addressing current legal charges or preventing the risk of future domestic violence charges
**Improving Our Health as a Couple—**Learning ways we can improve the overall health of our relationship
**Reducing Our Risk for Future Health Problems—**Learning how relationships are connected to health problems like cardiovascular disease
**Improving Your Sexual Health—**Reviewing strategies to improve your sex life
**Improving Intimacy Between You and Your Partner—**Identifying ways to improve sexual intimacy
**Rekindling love—**Finding ways to improve positive emotions in a relationship
**Relationship Evaluation—**Getting a professional assessment on whether there are any problem areas in my relationship that could lead to future conflict or divorce
** Is it Time to End?—**Reviewing strategies to identify if it is time to end a relationship

Note. Items marked with an asterisk (*) were merged in analysis due to low endorsement of each individual item (i.e., at least one item < 10) and similarity between their descriptions.

**Table 2:** Participant Characteristics by Intimate Partner Violence (IPV) Risk Group.

	*n* (%)/M(SD)	*n* (%)/M(SD) by IPV Risk Group			Group Differences	
Characteristic Level	Full Sample (*n* = 299)	No IPV (*n* = 84)	Verbal Only (*n* = 152)	Severe IPV (*n* = 63)	χ^2^/F (*df*)	*p*
Female	177 (59%)	53 (63%)	85 (56%)	39 (62%)	1.4 (2)	0.50
Age	50.07 (13.46)	54.07 (13.96) ^A^	49.34 (13.25) ^B^	46.42 (12.03) ^B^	**6.45 (2295)**	**0.002**
Lesbian/Gay/Bisexual	40 (13%)	13 (15%)	14 (9%)	13 (21%)	5.46 (2)	0.65
Married (vs. Dating)	248 (83%)	73 (87%)	124 (82%)	51 (81%)	1.31 (2)	0.52
Cohabitating (vs. Living Apart)	280 (94%)	76 (90%)	145 (95%)	59 (94%)	2.2 (2)	0.33
Hispanic/Latino	15 (5%)	6 (7%)	5 (3%)	4 (6%)	2.18 (2)	0.34
Race					2.51 (4)	0.64
White	263 (88%)	76 (90%)	133 (88%)	54 (86%)		
Black	18 (6%)	4 (5%)	11 (7%)	3 (5%)		
Other	18 (6%)	4 (5%)	8 (5%)	6 (10%)		
Mental Health Screens:						
Depression	107 (36%)	27 (32%) ^A^	48 (32%) ^A^	32 (51%) ^B^	**7.83 (2)**	**0.02**
Thoughts of Self-Harm	45 (15%)	10 (12%)	22 (14%)	13 (21%)	2.23 (2)	0.33
Post-Traumatic Stress Disorder	95 (32%)	21 (25%)	47 (31%)	27 (43%)	5.40 (2)	0.07
Alcohol Use Disorder	36 (12%)	5 (6%)^A^	17 (11%)^A^	14 (22%)^B^	**9.21 (2)**	**0.01**
Relationship Health Measures:						
Use of Verbal Conflict	6.23 (8.39)	---	7.54 (8.17) ^A^	10.43 (9.80) ^B^	**6.40 (1213)**	**0.01**
Experience of Verbal Conflict	6.20 (8.50)	---	7.84 (8.75)	9.56 (9.22)	2.71 (1213)	0.10
Low Satisfaction	121 (40%)	24 (29%) ^A^	61 (40%) ^A^	36 (57%) ^B^	**12.21 (2)**	**0.002**
IPV Experience Screen	152 (51%)	12 (14%) ^A^	87 (57%) ^B^	53 (84%) ^C^	**71.53 (2)**	**<0.001**
Fear/Control by Partner	47 (16%)	6 (7%) ^A^	20 (13%) ^A^	21 (33%) ^B^	**20.17 (2)**	**<0.001**
At Risk of Injury by Partner	26 (9%)	5 (6%) ^A^	6 (4%) ^A^	15 (24%) ^B^	**23.24 (2)**	**<0.001**

Notes: To detect meaningful trends, we followed significant tests of group differences (bolded) with pairwise comparisons of each risk group. Different superscripted letters (e.g., A vs. B) represent statistically significant differences (*p* < 0.05). No IPV = Denied last year use or experience psychological IPV, physical IPV, sexual IPV, or injury. Verbal Only = Participants reported last year use or experience of mild psychological IPV (yelling/insults) but denied other forms of IPV. Severe IPV = Participants reported last year use or experience severe psychological IPV, physical IPV, sexual IPV, or injury.

**Table 3:** Preferences for Treatment Attributes by Intimate Partner Violence (IPV) Risk Group.

	*N* (%) in Full Sample (*n* = 299)	*n* (%) Selecting Each Choice by IPV Risk Group			Group Differences	
Aattribute Choice		No IPV (*n* = 84)	Verbal Only (*n* = 152)	Severe IPV (*n* = 63)	χ^2^ (*df*)	*p*
Preferred Type		A	B	B	**20.67 (8)**	**0.008**
Couple	**110 (37%)**	**23 (27%)**	**55 (36%)**	**32 (51%)**		
Individual	84 (28%)	**23 (27%)**	46 (30%)	15 (24%)		
Virtual health coach	45 (15%)	15 (18%)	19 (13%)	11 (17%)		
Group OR Class	11 (4%)	0 (0%)	9 (6%)	2 (3%)		
No type preference	43 (14%)	19 (23%)	21 (14%)	3 (5%)		
Preferred Format					13.95 (10)	0.175
Face-to-face	**168 (56%)**	**45 (54%)**	**85 (56%)**	**38 (60%)**		
Internet	27 (9%)	4 (5%)	16 (11%)	7 (11%)		
Phone OR Video Chat	22 (7%)	3 (4%)	13 (9%)	6 (10%)		
Self-help materials	20 (7%)	7 (8%)	10 (7%)	3 (5%)		
Mobile app	19 (6%)	5 (6%)	13 (9%)	1 (2%)		
No format preference	37 (12%)	16 (19%)	13 (9%)	8 (13%)		
Preferred Location		A	A,B	B	**23.03 (8)**	**0.003**
VA Primary Care	**78 (26%)**	**22 (26%)**	**43 (28%)**	13 (21%)		
Non-VA facility	68 (23%)	11 (13%)	35 (23%)	**22 (35%)**		
VA Behavioral Health	39 (13%)	7 (8%)	20 (13%)	12 (19%)		
Vet Center	22 (7%)	4 (5%)	12 (8%)	6 (10%)		
No location preference	84 (28%)	35 (42%)	40 (26%)	9 (14%)		
Preferred Duration					13.89 (10)	0.178
1 visit	36 (12%)	9 (11%)	25 (16%)	2 (3%)		
2–3 visits	**72 (24%)**	**16 (19%)**	**39 (26%)**	**17 (27%)**		
4–6 visits	**77 (26%)**	**19 (23%)**	**40 (26%)**	**18 (29%)**		
7–12 visits	30 (10%)	7 (8%)	15 (10%)	8 (13%)		
13+ visits	13 (4%)	3 (4%)	6 (4%)	4 (6%)		
No duration preference	64 (21%)	25 (30%)	26 (17%)	13 (21%)		

Notes. See Table 1 for non-technical descriptions provided to respondents for Preferred Type and Format. Preferred Location and Duration items and responses presented verbatim. Response(s) with highest endorsement bolded for ease of interpretation. Responses with low endorsement (*n* < 10) were merged with similar categories as denoted by an “OR.” Percentages may not total to 100% due to missing/blank responses. Significant tests of group differences (bolded) were followed by chi-squared comparisons for each pair of risk groups. Different column headers (e.g., A vs. B) represent satistically significant differences in response patterns. No IPV = Denied last year use or experience psychological IPV, physical IPV, sexual IPV, or injury; Verbal Only = Participants reported last year use or experience of mild psychological IPV (yelling/insults) but denied other forms of IPV. Severe IPV = Participants reported last year use or experience severe psychological IPV, physical IPV, sexual IPV, or injury as a result of a conflict.

**Table 4:** Participants Likely to Attend Treatments Addressing Specific Concerns by Intimate Partner Violence (IPV) Risk Group.

	*n* (%) Likely to Attend in Full Sample (N = 299)	*n* (%) Likely to Attend by IPV Risk Groups			Group Differences	
How Likely Would You be to Attend Help Focused on…		No IPV (*n* = 84)	Verbal Only (*n* = 152)	Severe IPV (*n* = 63)	χ^2^ (2)	*p*
Improving Our Health as a Couple	**172 (58%)**	39 (46%)	**90 (59%)**	**43 (68%)**	6.04	0.05
Improving Our Relationship	**158 (53%)**	39 (46%)	**77 (51%)**	**42 (67%)**	5.81	0.05
Reducing our Risk for Future Health Problems	**154 (52%)**	37 (44%)	**81 (53%)**	**36 (57%)**	2.07	0.36
Improving Couple Communication	**152 (51%)**	39 (46%)	74 (49%)	**39 (62%)**	3.46	0.18
Rekindling Love	**151 (51%)**	38 (45%)	73 (48%)	**40 (63%)**	5.31	0.07
Learning How to Be Calm	141 (47%)	34 (40%)	71 (47%)	**36 (57%)**	3.38	0.18
Improving your Sexual Health	135 (45%)	35 (42%)	63 (41%)	**37 (59%)**	5.39	0.07
Improving Intimacy between You & Your Partner	135 (45%)	36 (43%)	62 (41%)	**37 (59%)**	5.80	0.05
Relationship Evaluation	112 (37%)	22 (26%) ^A^	58 (38%) ^A,B^	**32 (51%) ^B^**	**8.31**	**0.02**
Anger Management	103 (34%)	25 (30%)	49 (32%)	29 (46%)	4.42	0.11
Improving the Home for My Children	91 (30%)	25 (30%) ^A,B^	38 (25%) ^A^	28 (44%) ^B^	**8.03**	**0.02**
Is It Time to End?	75 (25%)	16 (19%) ^A^	34 (22%) ^A^	25 (40%) ^B^	**8.75**	**0.01**
Improving Safety in a Relationship	47 (16%)	13 (15%) ^A,B^	16 (11%) ^A^	18 (29%) ^B^	**10.82**	**0.004**
Reducing Legal Risk	41 (14%)	14 (17%)	15 (10%)	12 (19%)	4.41	0.11

Notes. Each topic area rated on a Likert scale rating likeliness of attending a service from 1 (*Very Unlikely*) to 5 (*Very Likely*). Each row represents the count of participants who selected they were *Likely* or *Very Likely* to attend a service focused on that topic. Concerns with >50% endorsement (i.e., a majority of participants reporting they are likely to attend) bolded for ease of interpretation. See Table 1 for full descriptions provided with each concern. To detect meaningful trends, we followed significant chi-square tests of group differences (bolded) by chi-squared comparisons of each group. Different superscripted letters (e.g., A vs. B) represent significant differences in proportion likely to attend (*p* < 0.05). No IPV= Denied last year use or experience psychological IPV, physical IPV, sexual IPV, or injury. Verbal Only = Participants reported last year use or experience of mild psychological IPV (yelling/insults) but denied other forms of IPV. Severe IPV = Participants reported last year use or experience severe psychological IPV, physical IPV, sexual IPV, or injury.

## Data Availability

All study protocols, measures, and a full annotated syntax file of all analyses conducted for this manuscript can be obtained by contacting the corresponding author. Deidentified data are available from the senior author on reasonable request.
